# Interactive Cognition of Self-driving: A Multidimensional Analysis Model and Implementation

**DOI:** 10.34133/research.0903

**Published:** 2025-12-02

**Authors:** Nan Ma, Zhixuan Wu, Kai Li, Genbao Xu, Qin Xia, Cheng Xu

**Affiliations:** ^1^ School of Information Science and Technology, Beijing University of Technology, Beijing 100124, China.; ^2^ School of Computer Science, Beijing University of Posts and Telecommunications, Beijing 100876, China.; ^3^ Dongfeng USharing Technology Co., Ltd., Wuhan 430056, China.; ^4^ China Automotive Institute Vehicle City Integration (Wuhan) Technology Co., Ltd, Wuhan 430078, China.; ^5^ Beijing Key Laboratory of Information Service Engineering, Beijing Union University, Beijing 100101, China.

## Abstract

Self-driving vehicles rely closely on interactions with humans, vehicles, and the surrounding environment. However, the interactive analysis of self-driving is impacted by multiple perception sources, heterogeneous data, and complex environments in actual scenes. Due to the above issues, we are often unclear about the behavior of self-driving vehicles and do not understand their decisions, and it is also difficult to achieve synergy with our human intentions. We introduce the significance of research in the field of self-driving interactive cognition, detailing its components and underlying infrastructure. Furthermore, we demonstrate how self-driving interactive cognition, inspired by the Wiener model, embodies intelligence in complex environments with the purpose of stressing the importance of interactive cognition in complex environments and scientifically evaluating the analysis of machine interactive cognition. Then, a multidimensional analysis model of self-driving interactive cognition is established based on perceptual information acquisition, multichannel and cross-modal data registration, attention mechanism, visual recognition and understanding, and embodied dynamic control. Supported by the above, we build a multiview spatiotemporal graph convolutional network (MV-STGCN) model for action recognition to realize vehicle-to-human body language interactive cognition. Most importantly, we innovatively propose a nonlinear-CRITIC–technique for order preference by similarity to an ideal solution (TOPSIS)-based method to analyze the interactive cognition analyses of different action recognition algorithms efficiently, such as MV-STGCN. Future self-driving vehicles are bound to demonstrate multichannel and cross-modal intelligence perception and human–vehicle-friendly interaction, and we are committed to better realizing humanoid driving analysis and the embodied intelligence of self-driving vehicles. “Self-driving + interactive cognition” could make future vehicles become interactive wheeled robots that can be trusted and better serve human society.

## Introduction

Self-driving vehicles have profoundly changed the modes of transportation used by people, the intelligence level of transportation, and even production and life in the future. Researchers are primarily concerned with the driving skills and techniques of self-driving systems in vehicles [1]. However, the abundant interactive cognitive analytic abilities that a driver must possess before being issued a license are often neglected. For example, if someone waves to the vehicle on the sidewalk of the road, it might mean “pulling over”; if someone raises his hands at the crosswalk, it could mean “please go”; and if a driver waves through the window, it might mean asking the other party to go first to avoid deadlocks. These interactions need to be effectively and efficiently recognized by the self-driving system and remain stable across different nations, regions, and cultures. In addition, it needs to satisfy the interactive cognition demands of evolving human beings eventually.

Markkula et al. [2] and Rummel [3] proposed that interaction is an attempt to influence or interpret each other’s subjective experiences or intentions. For example, an agent analyzes other agents’ actions and makes decisions based on the resulting influence. Li et al. [4] and Fan et al. [5] constructed natural human–computer interaction models to explain the action of humans interacting with dynamic tasks and predict human intention. They proposed that human–machine interaction and artificial intelligence (AI) have the same research objectives and objects and are complementary and mutually reinforcing relationships. The development of AI will constantly break through and innovate human–machine interaction and eventually drive the development of human–machine interaction. Yan et al. [6] and Huang et al. [7] constructed a computational framework for understanding natural interaction intention to obtain accurate interaction intentions based on behavioral data as an efficient method of semantic information exchange between humans and machines. Xue et al. [8] established hierarchical representations of complicated traffic scenes using multisensor datasets to produce decisions for self-driving vehicles and achieve accurate perceptions of traffic scenes. Ma et al. [9] proposed self-driving vehicles’ interactive cognition and evolution growth in uncertain and complex environments. In this paper, we delve into the field of autonomous driving interaction cognition, focusing on building more accurate interaction cognition models and achieving efficient integration of multimodal perception and decision-making.

The efficient operation system of self-driving vehicles has to establish a bidirectional interaction with humans, vehicles, and roads in actual traffic, generating interactive intelligence. The “Intelligent Vehicle Innovation and Development Strategy” [10] emphasizes the importance of intelligent decision control, the perception of complex environments, human–machine interaction, human–machine co-driving, vehicle–road interaction, etc. Intelligent interaction is the dominant technology for self-driving vehicles. Because interactions are a common cognition form in human society, interactive cognition is an important guarantee of human–machine behavioral synergy. Interactive cognition is the process of enhancing machine intelligence and facilitating human–machine integration by constructing intelligent expressions and learning methods that are unified with the physical world, through technologies such as cross-model perception, machine learning, and cognitive computing.

“Self-driving + interactive cognition” could make future vehicles become interactive wheeled robots that can be trusted and better serve human society. AI is a core technology for self-driving. With the commercialization of self-driving, lane tracking, flow monitoring, and lane-changing techniques have been widely applied. Do self-driving vehicles have a clear understanding of human behavior during driving? Can humans remain informed of timely decisions? We need to answer these questions regarding human and vehicle coordination. Therefore, it is necessary to make self-driving more knowledgeable about “human sophistication”, interactive, learning, and trustworthy [11,12]. In response to this lack of interaction awareness, self-driving vehicles should independently cope with the diverse uncertainties of an episodic nature that are often encountered during the driving process. The difficulties in their development go beyond the nature of vehicle dynamics and the variety of sensors required. It is also necessary to simulate the cognitive ability to achieve self-driving prediction and control of the driver in the circuit, with a driving license. Moreover, it builds up driving skills through online learning. It makes it possible to personalize driving by having both a rich memory for the prevention and disposal of driving accidents and the ability to interact with different humans or requests such as owners, occupants, operation and maintenance staff, developers, and remote service requests. Therefore, personification is a key aspect of realizing self-driving, meaning self-driving vehicles must have sufficient interactive cognition analyses for road safety and be acceptable by society.

To tackle these challenges, we present a novel multidimensional interactive cognition analysis model; our main contributions are summarized below:

• We propose an interactive cognitive ability model for self-driving vehicles based on multidimensional cross-modal data processing, selective attention mechanism, and visual perception, which includes perceptual information acquisition, multichannel and cross-modal data registration, attention mechanism, visual recognition and understanding, and embodied dynamic control.
• We propose a self-driving interactive cognition inspired by the Wiener model that enhances intelligent decision-making and adaptability in complex driving environments of human–vehicle–road coordination.
• We innovatively propose a nonlinear-CRITIC–technique for order preference by similarity to an ideal solution (TOPSIS)-based method to analyze the interactive cognition analyses of different action recognition algorithms efficiently, optimizing performance analysis in interactive cognition systems.

### Composition and infrastructure of self-driving interactive cognition

In the Turing test for self-driving vehicles, intelligent vehicles will definitely exhibit multichannel and cross-modal perceptual interactions, and it will be difficult for people to tell the difference between machine drivers and human drivers. In this section, we delve into the constituent elements of unattended driving interaction cognition, as well as the necessity and challenges of constructing its underlying infrastructure. By analyzing these core concepts, we can better understand how unattended vehicles can ensure traffic safety and efficiency while embodying more intelligent interaction cognitive abilities.

**Fig. 1.**
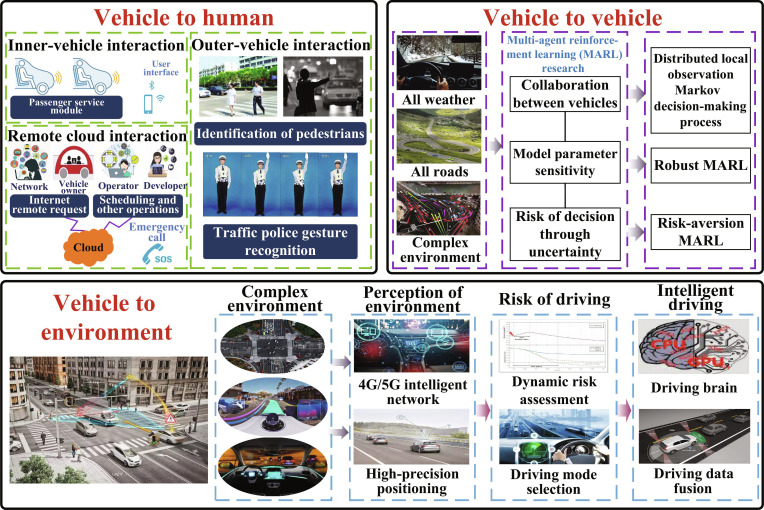
Composition and infrastructure of self-driving interactive cognition.

According to the different cognitive objects of self-driving vehicles, the interactions can be divided into 3 categories: vehicle-to-human, vehicle-to-vehicle, and vehicle-to-environment interactions [9]. As shown in Fig. 1, vehicle-to-human interaction involves the self-driving vehicles’ understanding of the gestural behavior of humans around it, which requires solving the difficulties of multiple scene objects, confusion, and obscuration as well as high recognition speed requirements. For example, in order to obtain multiview visual data using onboard sensors, a multistage and multibranch convolutional neural network (CNN) structure is built to extract data features. After coding the features for depth, a recurrent neural network is applied to recognize dynamic gestures in self-driving vehicles based on temporal relationships and combined with the attention mechanism to effectively improve recognition efficiency. In the context of interactive cognition between autonomous vehicles and humans, it is necessary to understand and predict the posture of pedestrians and the gestures of traffic police, among other interactive behaviors, and make judgments about specific situations to make decisions such as stopping or turning.

Vehicle-to-vehicle interaction is important for road safety, stability, comfort, and energy efficiency. In view of the diverse, time-varying, and uncertain problems of typical driving scenes such as overtaking, lane change, and meeting narrow roads, risk suppression heuristic algorithms adapt to changes in unknown scenes to avoid potential dangers or conflicts, and the multi-agent reinforcement learning algorithm can address key issues such as collaboration between vehicles, and it makes the machine achieve humanlike safe driving by realizing transfer learning in new environments via setting up autonomous reward and punishment functions.

Vehicle-to-environment interaction needs to infuse the 4G/5G sensing data in the smart grid and improve the sensing network coverage for Vehicle to Everything (V2X) in the road network. It adapts to complex traffic environments and changing driving conditions via traffic information collection, rapid positioning, and intervehicle collaboration. Extensive data training combined with prior knowledge [13,14] can continuously improve the self-driving vehicle’s analyses of embodied control, so as to better interact with humans, surrounding vehicles, and environments and enhance the analysis of humanlike driving.

## Results

In this paper, we introduce the process in multidimensional analysis model verification of self-driving interactive cognition. Because body language interactions between vehicles and humans have become one of the key issues in the field of self-driving cognition, we take the interaction between self-driving vehicles and humans as an example to analyze the establishment and evaluation. During the driving process of self-driving vehicles, pedestrian postures and traffic police gestures are typical interaction modes between vehicles and humans. It is necessary to recognize and understand people’s behavioral intentions and then make decisions such as parking and turning, so as to achieve body language interaction between vehicles and humans. Unfortunately, real scenes often face changes in weather conditions such as strong road lighting, shadows, wind, rain, fog, snow, and haze and may also encounter complex road conditions such as blocking by obstacles, objects moving rapidly and instantaneously, and numerous pedestrians at road intersections, which make it difficult for self-driving vehicles to recognize people’s body language timely and accurately and make it hard for vehicles to make accurate decisions.

To enhance the interactive cognition capabilities of autonomous vehicles in real-world driving scenarios, we have established a self-collected multiview pedestrian action and traffic police gesture dataset that covers some conditions that autonomous vehicles may encounter, such as different times of day, weather conditions, human behaviors, and distances between vehicles and pedestrians. This comprehensive approach ensures that the vehicle’s perception system is robust and can accurately interpret and respond to the complex interactions that occur in traffic environments. The NTU RGB+D dataset provides a diverse range of everyday human behaviors, which helps in understanding general human actions that may be relevant in traffic scenarios. On the other hand, the Chinese police gesture dataset focuses on specific gestures directly related to traffic control. By integrating these datasets, the analysis can account for both general human behavior and specific traffic-related gestures, thereby expanding the scope of interactive cognition for autonomous vehicles.

### Multichannel and cross-modal data acquisition and processing for vehicles and humans

#### Self-collected multiview pedestrian action and traffic police gesture dataset

**Fig. 2.**
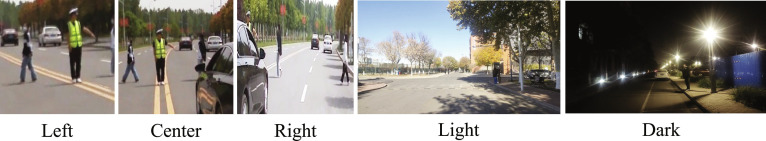
An example diagram of a self-collected multiview dataset.

To facilitate multiview image-based action recognition, we construct a self-collected multiview dataset focusing on pedestrian actions and traffic police gestures, as shown in Fig. 2. The dataset covers 6 dimensions of diversity: time of day, weather conditions, participant variability, action categories, distances between vehicles and humans, and multiview camera perspectives. The data acquisition devices operate at a frame rate of 30 frames per second. In total, the dataset contains 72,450 pedestrian action images and 250,760 traffic police gesture images, with 70% used for training and 30% for validation. To closely emulate the perception setups commonly adopted in autonomous driving systems, we mount 3 synchronized cameras on the front of the vehicle, positioned at left, center, and right viewpoints. This configuration mirrors the typical multicamera fusion systems used in commercial autonomous driving platforms, where the cameras are spatially aligned and temporally synchronized to capture complementary and overlapping fields of view with slight angular offsets. We collect multiview data of pedestrians, roadside taxis, and traffic police gestures in the traffic scenes from 3 different distances and perspectives under different light intensities and weather conditions. The dataset is available at http://www.mananlab.tech/datasets. The dataset naming conventions are set as follows:

• Scene: S (simple) and C (complex) represent simple or complex scenes with vehicles, respectively.
• Angle of view: L (left), R (right), and C (center) respectively represent the left, right, and middle angles of view.

For example, SL001 indicates the pedestrian action data from the left view in a simple scene, CR001 indicates the pedestrian action data from the right view in a complex scene, 001 indicates the first video of the action, and SL001_1 indicates the first frame of the video.

#### Public datasets

NTU RGB+D [15]: The NTU RGB+D dataset stands as a cornerstone in the field of human action recognition, offering an extensive and diverse collection of actions captured for research purposes. There are 60 types of behaviors in the dataset, including drinking water, sitting down, and standing up, with a total of 56,880 samples. The samples were carried out by 40 volunteers with 3 different camera perspectives. Each example contains one action and is guaranteed to have up to 2 targets, captured simultaneously by 3 Microsoft Kinect v2 cameras from different views. The authors of this dataset recommend 2 benchmarks: (a) Cross-subjects (CS): training data come from 20 subjects, and testing data come from 20 others. (b) Cross-view (CV): training data come from camera views 2 and 3, and test data come from camera view 1. The NTU RGB+D dataset’s diversity and comprehensiveness make it well suited for a wide range of applications. Additionally, the dataset’s rich annotation and multimodal data offer opportunities for exploring advanced research areas such as skeleton-based action recognition, depth-based feature extraction, and multiview fusion techniques.

Chinese police gesture dataset: It contains 20 videos of people making traffic police gestures, including 8 kinds of traffic police gestures, namely, stop, straight, turn left, turn left wait, turn right, change lane, slow down, and pull over, annotated frame by frame. The total video length of the dataset is about 2 h, a shot of different cohorts of police in uniforms and different scenes using different devices.

### Verification of self-driving interactive cognition

**Fig. 3.**
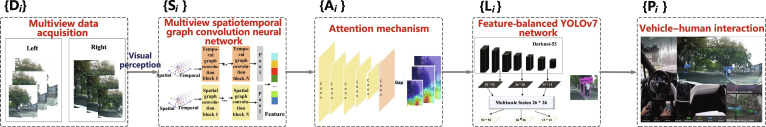
A situational awareness model of vehicle-to-human body language interaction.

This study adopted the vehicle-to-human body language interactive cognition model to address the challenges in perception analysis and lack of data information in interactive cognition, as shown in Fig. 3. This model was used to obtain complete information on the target data and effectively improve the accuracy of target recognition. First, multiview data $\left\{D_{i}\right\}$ were obtained from 2 or more input perspective data. Subsequently, we adopted a multiview spatiotemporal graph convolutional network (MV-STGCN) and introduced the attention mechanism for the feature information of important nodes. Feature extraction was performed on the salient areas to recognize human postures and gestures and further improve the feature recognition ability. Based on the NTU RGB+D dataset and gesture dataset of the Chinese traffic police, problems such as the poor detection effect and timeliness of traffic police gestures in complex traffic scenes were solved. We extracted the features of the targeted candidate region through a spatial–temporal graph CNN. We fully captured the temporal and spatial features of the candidate region in the video. They can be used to accurately and efficiently detect the established tasks of the traffic scenes. We used human joints and common groups to model the graphical structure. The features of each node are composed of coordinating information and detection confidence. Specifically, for the NTU RGB+D dataset, the common feature is expressed as (*x*, *y*, *z*). The articular group is characterized by multiple joints. Suppose that a common group $v_{\mathrm{ab}}$ contains subjoints $v_{a}$ and $v_{b}$; then, the characteristics of graph nodes composed of joint groups can be described by the following formula:

$$f\left(v_{a}\right)=\left(x_{1},\;y_{1},\;z_{1}\right)$$ (1)

$$f\left(v_{b}\right)=\left(x_{2},\;y_{2},\;z_{2}\right)$$ (2)

$$f\left(v_{\mathrm{ab}}\right)=\left\{\left(x_{1},\;y_{1},\;z_{1},\;x_{2},\;y_{2},\;z_{2},\;C\right)|\;v_{a},\;v_{b}\in V_{0}\right\}$$ (3)

Temporal feature extraction network and spatial feature extraction branches can focus on mining the temporal and spatial information, respectively. The input and output features in the temporal and spatial feature extraction network branches are independent. We described the process as follows:

$$f_{\mathrm{tem}\_\text{out}}^{i}=\mathrm{Tem}\_\mathrm{GCN}\left(f_{\mathrm{tem}\_\text{in}}^{i}\right)$$ (4)

$$f_{\mathrm{spa}\_\text{out}}^{i}=\mathrm{Spa}\_\mathrm{GCN}\left(f_{\mathrm{spa}\_\text{in}}^{i}\right)$$ (5)

where $f_{\mathrm{tem}\_\text{in}}^{i}$ and $f_{\mathrm{spa}\_\text{in}}^{i}$ represent the input features of the *i* graph convolution block in the branch of the temporal and spatial feature extraction networks, respectively, and the parameter configurations of the time and space features are different. Tem_GCN and Spa_GCN represent the graph convolution blocks of the temporal and spatial feature extraction network branches, respectively. $f_{\mathrm{tem}\_\text{out}}^{i}$ and $f_{\mathrm{spa}\_\text{out}}^{i}$ represent the output feature $S_{i}$ of the *i*th graph convolution block in the branch of the temporal and spatial feature extraction networks, respectively.

We introduced an attention mechanism to extract salient features from the entire input video data. Specifically, 64 × 64 dimensional feature vectors of *K* clusters were obtained through clustering. The *K* cluster centroids are selected from $S_{i}$ and denoted as $m_{j}\;\left(j=1,\;2,\;\ldots ,\;k\right)$, where $m_{j}$ stands for the center of mass of the *j*th class. We used the Euclidean-style distance calculation formula and the moving edge detection method to obtain the motion significance mapping $S_{i^{\prime }}$, and then the overlapping motion significance mapping is continuously superimposed. Finally, the significance value $\mathrm{SV}\left(i,j\right)$ of each mapping map is obtained cumulatively as the weight value W of the mapping map in region D, where $\left(i,\;j\right)$ is the coordinate value of mapping map $S_{i}$. The attentional weight vector $A_{x,y}^{t}$ of the final motion saliency graph can be expressed as

$$A_{x,y}^{t}=\tanh\left(W_{i,j}h_{t-1}+f_{x,y}^{t}\right)$$ (6)

where $h_{t-1}$ is the hidden state of the long short-term memory (LSTM) unit at time *t* − 1, $W\left(i,\;j\right)$ represents the weight value of position $\left(i,\;j\right)$, and $f_{x,y}^{t}$ is the output vector of position $\left(x,\;y\right)$ at time *t*.

We proposed the feature-balanced YOLOv7 network (BYOLO) [16] to improve pedestrian detection accuracy in complex scenes. We used the same resolution to integrate high-level semantic and low-level detail features, learn semantic features at different levels, and obtain behavior recognition results $L_{i}$ to realize vehicle–human interaction $P_{i}$.

### Analysis verification of self-driving interactive cognition

ResNet50 pre-trained on ImageNet was used as the backbone network for feature extraction. In order to extract features more fully, the size of all video frames was changed to 224 × 224, and different frame sampling combinations were used for each video in different training cycles. The batch size was set to 8, the epoch was 200, the learning rate was set to 1 × 10^−7^, and step attenuation was performed after the 30th and 50th epochs. The decay rate was 0.1. In this experiment, a sliding window was set, and 40 frames were extracted as a sample for training. Multiview data $D_{i}$ were obtained, and the available data reached 99%. Spatiotemporal feature $S_{i}$ was obtained by the human body point feature extraction method based on the spatial–temporal graph convolution neural network, with an accuracy of 96.8%.

**Fig. 4.**
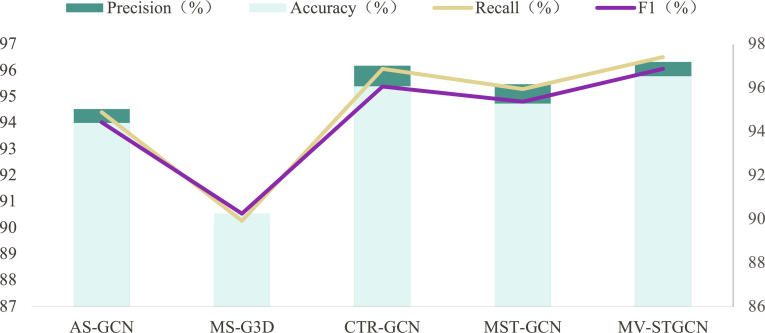
Comparisons of different methods on the imitating multiview police gesture dataset in terms of accuracy, precision, recall, and F1.

Finally, feature learning was carried out by the feature-balanced YOLOv7 network model to obtain the final result $L_{i}$, which was compared with the most advanced method in the NTU RGB+D dataset, as shown in Table 1. Our proposed model multiview spatial–temporal graph convolution network (MV-TSHGNN) achieved 96.1% (CV) and 91.2% (CS) accuracy on the NTU RGB+D dataset, Action Capsules [17] exhibited a competitive performance with 96.3% in CV and 90.0% in CS, closely followed by MV-IGNet [18], which achieved 96.1% in CV and 88.8% in CS. These results highlight MV-TSHGNN’s competitive performance, particularly in the CS setting, where it outperforms methods like ST-GCN (88.3% CV and 81.5% CS) and AS-GCN (94.2% CV and 86.8% CS). These comparisons demonstrate that our model, MV-TSHGNN, provides superior accuracy in CS performance, thus affirming its effectiveness in capturing complex spatiotemporal features for human action recognition in the NTU RGB+D dataset. As shown in Fig. 4, we experimented on the imitating multiview police gesture dataset, where different GCN methods were evaluated and compared with respect to accuracy, precision, recall, and F1 score. MV-STGCN demonstrated the highest performance across all metrics, achieving an accuracy of 96.53%, a precision of 96.32%, a recall of 96.52%, and an F1 score of 96.88%. These results highlight the effectiveness of MV-STGCN in handling multiview data and its capability to extract comprehensive spatiotemporal features, which are critical for accurately recognizing complex gestures from multiview.

**Table 1.** Comparisons with different methods on the NTU RGB+D dataset in terms of accuracy (%). The best result is highlighted in bold.

Method	Year	CV	CS
ST-GCN [34]	2018	88.3	81.5
PR-GCN [35]	2021	91.7	85.2
AS-GCN [36]	2019	94.2	86.8
MV-IGNet [18]	2020	96.1	88.8
2s-SDGCN [37]	2019	95.7	89.5
Action Capsules [17]	2023	96.3	90.0
LKA-GCN [38]	2023	96.1	90.7
4s Shift-GCN [39]	2020	96.5	90.7
**MV-STGCN**	2023	**96.1**	**91.2**

CV, cross-view; CS, cross-subjects; MV-STGCN, multiview spatiotemporal graph convolutional network

**Fig. 5.**
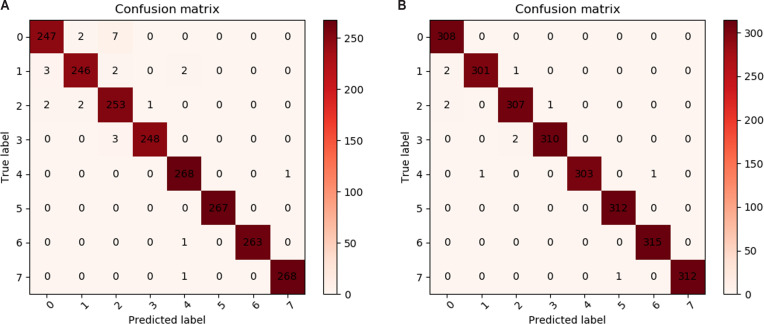
Visualization of MV-STGCN training results from the confusion matrix on the NTU RGB+D and imitating multiview police gesture datasets: (A) visualization of the MV-STGCN training result confusion matrix on the NTU RGB+D dataset (the first 7 actions) and (B) visualization of the MV-STGCN training result confusion matrix on the self-collected multiview police gesture dataset.

Experiment on the NTU RGB+D (CS) and imitating multiview police gesture datasets: We used the confusion matrix to analyze the reasons for misclassification on the NTU RGB+D and imitating multiview police gesture datasets. Figure 5 shows the data volume of various types selected during the model verification in this training, where the horizontal axis is the predicted result and the vertical axis is the real result. The experimental results show that the method has a higher recognition accuracy and fewer analysis errors.

**Fig. 6.**
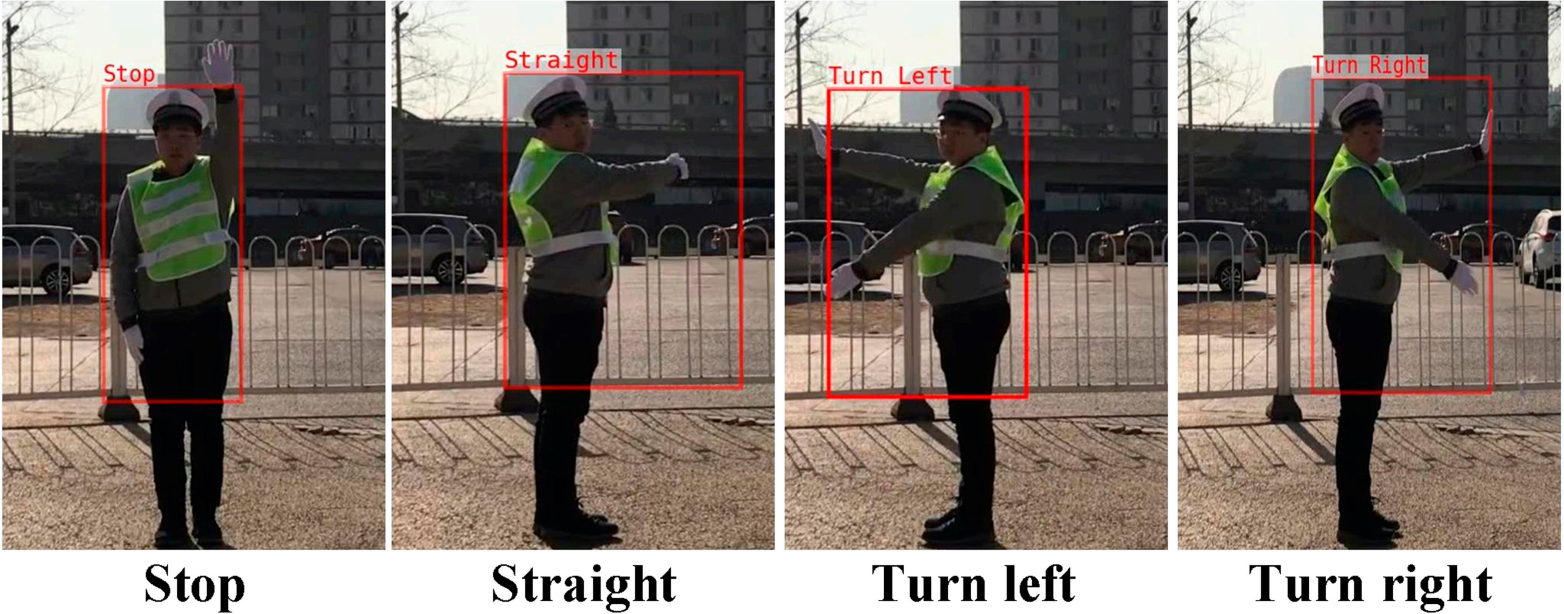
Chinese traffic police gesture dataset results.

The partial recognition results for the Chinese traffic police gesture dataset are shown in Fig. 6, highlighting the model’s performance across different gesture types.

In this experiment, we summarized the reliability and accuracy of the model. We used spatial and temporal features to obtain the frequent motion regions. Finally, the classification results are obtained by softmax. In conclusion, the temporal and spatial attention mechanisms can improve the interactive cognitive ability of vehicles and humans to a certain extent.

Taking the interactive cognition of body languages between vehicles and humans as an example, we analyzed 5 commonly used behavior recognition algorithms: MV-STGCN, ST-CNN, PKEN+LSTM, SlowFast, and ARCN for comprehensive evaluation and performance analysis. The cognitive assessment of the body language interaction between vehicles and humans has 8 dimensions, $D_{i}$, $S_{i}$, $A_{i}$, $L_{i}$, $C_{i}$, $P_{i}$, $V_{i}$, and $I_{i}$, and each dimension contains different indicators. We selected 12 indexes from different dimensions to evaluate different algorithms comprehensively. The specific assessment steps were as follows:

1. Determine the index set of vehicle–human body language interactive cognition capability.

$U=\left\{u_{1},\;u_{2},\;u_{3},\;u_{4},\;u_{5},\;u_{6},\;u_{7},\;u_{8},\;u_{9},\;u_{10},\;u_{11},\;u_{12}\right\}$, where $u_{1}$ to $u_{12}$ are the point cloud acquisition density, image frames, the average accuracy of cross-modal data matching, the absolute error of cross-modal data matching, the average accuracy of cross-source data matching, the absolute error of cross-source data matching, parameters in the spatial–temporal network model, the signal-to-noise ratio of image quality assessment, the mean square error of image quality assessment, accuracy for image recognition, recall for image recognition, accuracy for pedestrian action recognition, and the other 12 dimensions of the evaluation indicators, respectively.

2. Determine the evaluation result sets for different algorithms. $V=\left\{v_{1},\;v_{2},\;v_{3},\;v_{4},\;v_{5}\right\}$, where $v_{1}$ to $v_{5}$ correspond to MV-STGCN, ST-CNN, PKEN+LSTM, SlowFast, and ARCN, respectively.
3. Different algorithms obtain the original data matrix R according to the index set.

$$\begin{array}{c} R=\left(R_{1},\;R_{2},\;\ldots ,\;R_{5}\right)={\left(r_{\mathrm{ij}}\right)}_{5\times 12}=\\ \left(\begin{array}{cccccccccccc} 905 & 32 & 89.5 & 0.08 & 94.3 & 0.14 & 1.62 & 52 & 0.13 & 96.6 & 96.7 & 96.8\\ 862 & 30 & 86.1 & 0.22 & 89.6 & 0.34 & 1.87 & 48 & 0.25 & 91.5 & 92.1 & 91.7\\ 720 & 30 & 84.2 & 0.46 & 85.9 & 0.61 & 2.08 & 47 & 0.44 & 91.2 & 91.7 & 91.3\\ 685 & 28 & 82.9 & 0.54 & 83.3 & 0.82 & 2.32 & 47 & 0.51 & 91.0 & 91.4 & 90.5\\ 651 & 27 & 78.2 & 1.03 & 80.7 & 1.43 & 2.97 & 45 & 0.92 & 82.4 & 83.8 & 82.9 \end{array}\right) \end{array}$$ (7)

4. Standardize the original data matrix, calculate each indicator’s volatility S and conflict B, and obtain the information quantity D for each indicator.

$$S=\left(0.442\;0.389\;0.368\;0.384\;0.393\;0.386\;0.382\;0.369\;0.383\;0.360\;0.359\;0.358\right)$$ (8)

$$B=\left(6.721\;6.440\;8.064\;8.025\;6.352\;7.977\;8.028\;6.339\;8.035\;6.499\;6.465\;6.401\right)$$ (9)

$$D=\left(2.971\;2.511\;2.972\;3.084\;2.497\;3.081\;3.065\;2.343\;3.080\;2.340\;2.325\;2.295\right)$$ (10)

5. Calculate the weight matrix W of the body language interaction cognitive ability index between vehicles and humans according to the information of each indicator.

$$W=\left(0.091\;0.077\;0.092\;0.094\;0.076\;0.095\;0.094\;0.072\;0.095\;0.071\;0.069\;0.070\right)$$ (11)

6. According to 12 indexes of the 5 algorithms, the total evaluation score of different algorithms is calculated as C.

$$C=\left(0.275\;0.235\;0.199\;0.151\;0.140\right)$$ (12)

Self-driving relies on AI technologies such as sensing, data, and algorithms to obtain decision-making information, and the credibility and quantitative evaluation of these are crucial benchmarks for measuring the level of intelligent driving. The evaluation capability model plays a key role in autonomous driving systems, ensuring the reliability of decision-making processes based on sensors, data, and algorithms by analyzing the correlation between different metrics. The experimental validation shows that the MV-STGCN model achieved a comprehensive evaluation score of 0.275, which, when compared to the scores of the ST-CNN, PKEN+LSTM, SlowFast, and ARCN algorithms, not only confirms the performance of the MV-STGCN model but also highlights the importance of the evaluation capability model in enhancing and verifying the performance of autonomous driving technology. The application of such evaluation models not only provides a direction for future research but also helps to improve the design of self-driving systems, thereby enhancing their safety and reliability in practical applications. Furthermore, we analyze that autonomous vehicles may need to consider environmental factors in addition to the 12 aforementioned dimensions to understand human behavior in different contexts, which is derived from the above vehicle-to-human interaction experiments. For example, if someone waves to the vehicle from the roadside, it may signal “pull over”; if someone raises their hand at a crosswalk, it may mean “please proceed”. Therefore, significantly enhancing the interaction capabilities of autonomous vehicles can be achieved by understanding and effectively recognizing these behaviors.

We observed that our nonlinear-CRITIC–TOPSIS method developed in this paper provides a promising evaluative framework for action recognition models, such as MV-STGCN; it allows for a multidimensional evaluation that captures various facets of model performance, including perceptual data acquisition $D_{i}$, cross-modal data registration $S_{i}$, attention mechanism $A_{i}$, visual recognition and understanding $L_{i}$, embodied control $C_{i}$, and vehicle–human interaction $P_{i}$. This comprehensive assessment is crucial for action recognition models like MV-STGCN, which must perform well across different metrics to ensure reliable interactions in autonomous driving systems. It stands out for its ability to consider nonlinear relationships and correlations between performance indicators using Spearman’s rank correlation, assign objective weights based on data characteristics, provide a comprehensive assessment across various metrics, and adapt to diverse driving conditions to ensure the robustness and generalizability of the evaluated models.

## Conclusion

Self-driving vehicles must learn, evolve, and iteratively develop interactive cognition [19]. The analysis model of interactive cognition must effectively evaluate perceptual intelligence, behavioral intelligence, and learning analyses. The development of a self-driving intelligent interaction system that performs better in actual self-driving scenes, which is a multidimensional analysis model of self-driving interactive cognition, is established based on perceptual information acquisition, multichannel and cross-modal data registration, attention mechanism, visual recognition and understanding, and embodied dynamic control. The intelligent interactive research team conducted a series of studies and projects using the analysis model of interactive cognition to evaluate a self-driving intelligent interactive system. Since 2016, our team has worked closely with the Beijing Automotive Technology Center, Dongfeng USharing Technology Co., Ltd., Tianjin University, TRUNK, AIForceTech, and other universities and enterprises. We applied the proposed theory on the interactive cognition of self-driving and other intelligent interactive systems, which are adaptable to various scenarios and environments, across different sensors and on different vehicle platforms, facilitating intelligent interactions between vehicles and humans, as well as between vehicles. It was applied to the Dongfeng Sharing-VAN self-driving minibus, Zhongtong self-driving BUS, John Deere self-driving tractor, and China National Heavy Duty Truck HOWO TX/T5G self-driving truck among others. The Self-driving Cloud Intelligent Interactive System won the grand prize of the second China AI+ Innovation and Entrepreneurship Competition (over 2,000 teams participated). Research on the critical technologies of self-driving interaction will play a crucial role in the public recognition of self-driving vehicles. Future vehicles will also have machine intelligence with perception, cognition, behavior, interaction, learning, and self-growing, so as to better serve human society.

## Methods

The interactive cognition of self-driving is the realization of perceptual and behavioral intelligence. Self-driving should simulate people’s vision, auditory, and other external receptors to realize the perceptual intelligence of machines. At present, deep learning technology is mostly used to imitate human vision, auditory, and other perceptual organs. The behavioral intelligence of self-driving can be achieved through feedback embodied control, and behavioral intelligence is also known as embodied intelligence. For instance, in the case of a self-driving vehicle operating in urban traffic that is incapable of identifying the behavior and gestures of traffic police and passersby hailing a taxi, the vehicle will obviously not be allowed on the road under these circumstances. The self-driving vehicle has to show humanoid behavioral intelligence.

### The fusion of driving situations based on the right-of-way

Self-driving vehicles have great multichannel and cross-modal perceptual data in structured and unstructured forms, including position- and posture-aware data, images, videos, and point cloud data. Although these perceptual data are heterogeneous in structure, there is a semantic spatiotemporal association. The critical question of intelligent driving perception is how to realize the fusion of cross-modal heterogeneous perceptual data. GraphAlign [20] integrates the semantic segmentation features of images with 3-dimensional sparse CNN point cloud features from LiDAR by constructing nearest-neighbor relationships of point cloud features and utilizing projection calibration between images and point clouds to achieve cross-modality feature fusion. VirConvNet [21] employs the VirConv operator to reduce redundant computations with StVD and enhances the noise resistance of voxel features with NRConv, achieving efficient cross-dimensional feature fusion. TransFusion [22] enhances robustness against image degradation and sensor errors by adopting a relocalization fusion strategy from hard to soft associations. Its structure includes a convolutional backbone and a transformer-based detection head, where the decoder layers are responsible for predicting bounding boxes of LiDAR point clouds and adaptively fusing image features to leverage spatial and contextual information. Still, current challenges are as follows [23–25]: (a) Heterogeneous gap: Data with different models have different features, and accuracies may result in heterogeneous gaps. Although these perceptual data are heterogeneous with each other in content structure, there is spatiotemporal association semantically. Unifying heterogeneous perceptual data with different spatiotemporal accuracies, models, and structural features is critical in cross-modal fusion. (b) Semantic gap: There are a large variety of self-driving sensors, which have different noise characteristics, and each has its own advantages, resulting in perception errors, and conflict detection will inevitably occur. The resolution of semantic conflicts between multi-sensing data for trusted fusion between sensors by unifying intelligent expressions and semantic concepts is a challenging problem of cross-modal fusion.

**Fig. 7.**
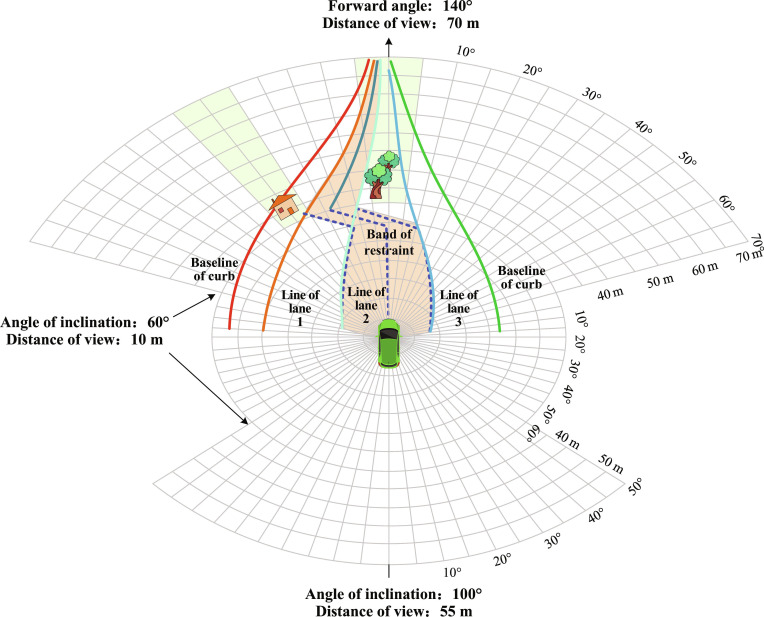
Right-of-way extraction in the log-polar driving situation chart [26].

In view of the heterogeneous and semantic gap existing in the cross-modal perception fusion of self-driving, our project team established a right-of-way-based cross-modal fusion method of driving situation under the guidance of the academician Li Deyi. The driving situation chart [26] is used as a unified template to infuse self-driving data, and heterogeneous sensing data with different spatiotemporal accuracies are projected onto a vehicle-centered log-polar driving situation chart, as shown in Fig. 7. Combined with driving priori knowledge, the sensor confidence network of different scenes is activated. The cross-modal heterogeneous sensing data are fused into driving right-of-way and converted into a driving cognitive arrow, realizing the unified expression of driving right-of-way as the semantics. This method enables effective spatiotemporal synchronization of heterogeneous data and resolves conflicts in cross-modal perception. It has been successfully applied in TRUNK autonomous container trucks and AIForceTech autonomous tractors.

### Self-driving interactive cognition inspired by the Wiener model realizes embodied intelligence

Researchers have accumulated a lot of achievements around the experimental design, algorithm design, and convergence proofs of intelligent systems, especially for the intelligent identification of linear systems. However, with the increasing number of scales and structures of self-driving interactive systems, the conventional method of approximately describing nonlinear processes based on linear models has the limitation of addressing the demand of high-precision control of self-driving. The implementation of self-driving interactive cognition involves not only the complexity and diversity of nonlinear models but also the uncertainty of models and the instability of data, which is a critical challenge we need to solve through self-driving interactive cognition. Therefore, how a self-driving vehicle better reflects the embodied intelligence of the vehicle, similar to manned driving, is a crucial component of the analysis model of self-driving interactive cognition.

Cognitive science studies demonstrate that human intelligence is acquired by interacting with actual scenes iteratively, and this acquisition method of intelligence is termed embodied intelligence [26,27]. Embodied intelligence refers to the ability of agents, such as wheeled robots, to perceive, understand, and act upon their environment through the interaction of algorithmically processed information with their physical form. Interaction plays a significant role in embodied intelligence. There are 3 core questions regarding how to interpret self-driving vehicle interactions: (a) From the perspective of vehicle cognition, how can the vehicle understand human behavior? (b) From the neurocognition perspective, what is the intrinsic correlation between semantic cognition and neurocognition in self-driving vehicles? (c) From the embodied cognition perspective, how can behavioral knowledge be transferred to self-driving systems?

The Wiener model [28] is a typical model of approximating nonlinear systems, where the input can be a typical physical transformation in the actuator, and the output can describe common sensor properties. Then, the feedback regulation control law of the identified Wiener model is designed to obtain the optimal control output. Feedback is the most effective mechanism to deal with open, complex, variable, and uncertain environments [29]. Therefore, self-driving interaction is able to describe the intelligence of a nonlinear process [29] based on the Wiener model, which can deal with uncertain environmental constraints and improve the smoothness of the embodied intelligence control of the self-driving system.

Norbert Wiener proposed to constitute the interaction between a machine and the environment by using feedback and control theory, realizing behavioral intelligence. The missions and actions of the machine are given by humans. Machines have perceptions, cognitions, and behaviors and are interactable, learnable, and self-growing. In order to improve the above analyses of machines, in an interactive environment, the state of the instantaneous, working, and long-term memory areas can be changed by interacting based on a complex set of complex actions with reward and punishment functions.

Machines are able to accomplish preset tasks through effective communication with humans. Human–machine interaction is the process by which humans teach and machines learn. In this way, machines can understand the tasks set by humans. However, deep learning is often simple and passive intelligence, lacking the language understanding of the mission given by humans. Only when the machine understands the mission given by humans via human–machine interaction, and is driven by the mission, can it achieve self-driving intelligence.

**Fig. 8.**
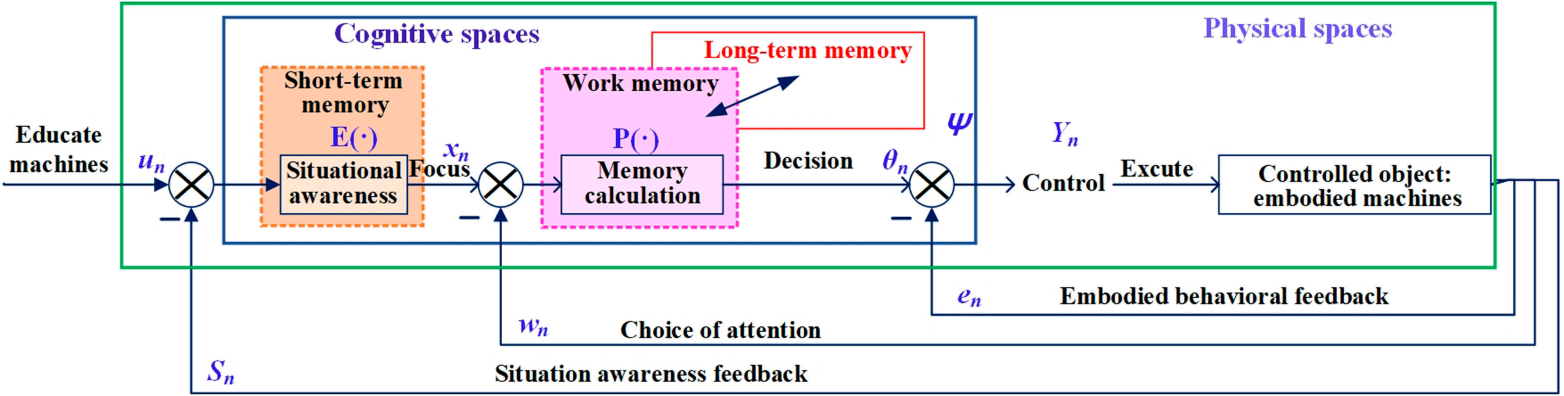
Schematic diagram of the interactive cognition of self-driving and learning feedback inspired by the Wiener model.

Intelligent machines continuously provide feedback and perform iterative optimizations on the environmental perception and behavioral processing capabilities of self-driving vehicles. By consistently collecting and analyzing interaction data between the vehicle and its environment, they enhance the adaptability and responsiveness to complex traffic scenarios [30], as shown in Fig. 8. Inspired by “how the feedback loop within the system changes the system itself” in the Wiener model, self-driving technology often pays too much attention to the environmental perception of signals and information processing in practical scenes while neglecting the interactive signals in the environment. For instance, self-driving vehicles perform analysis to perceive the indications of stop lines, signal lights, and traffic signs at a sophisticated intersection. However, at the peak period, the traffic police command on the spot adds uncertain information to the environmental variables in the model. We expect to fully consider the system feedback generated by perceptions of the environment and behavioral interactions to effectively improve the system’s capacity for decision-making and control.

We propose interactive cognition and learning feedback in self-driving inspired by the Wiener model, as shown in Fig. 8. The information feedback function of self-driving is shown in Eqs. (1) to (3). $u_{n}\;$ is the input agent task, $E\left(\cdot \right)\;$ is a short-term memory that generates the perceptual context, $x_{n}$ is the feature result of the perceptual information constrained by the sensor-aware machine learning behavior feedback $S_{n}$, and $P\left(\cdot \right)$ is the understanding and memory generated by working memory limited by long-term memory ψ [31]. More specifically, the agents constantly learn to understand the current tasks with existing knowledge and use the learned results to modify the present memory. $\theta_{n}$ is the attention selection feedback that constricts the behavioral performance $w_{n}$ as well as the agent’s understanding and memory of $P\left(\cdot \right)$. $Y_{n}\;$ is the output control instruction and is constrained by the embodied behavioral feedback $e_{n}$. The controlled object (agent) is finally controlled by the control command $\;Y_{n}$.

$$x_{n}=\left(u_{n}-s_{n}\right)\cdot E\left(\cdot \right)$$ (13)

$$\theta_{n}=\left(x_{n}-w_{n}\right)\cdot P\left(\cdot \right)$$ (14)

$$Y_{n}=\theta_{n}-e_{n}$$ (15)

The machine interacts with the instructor, receives instructional learning, and modifies long-term memory. The result of learning is memory, forming embodied intelligence at the end.

### Analysis model of self-driving interactive cognition

Inspired by the Wiener model, feedback is obtained for machine learning behaviors, attention selection, embodied actions, and other aspects of sensor perception, with the understanding that interaction is inseparable from feedback. This feedback lays the foundation for machine interactive cognition. We establish a multidimensional analysis model of self-driving interactive cognition based on perceptual information acquisition, multichannel and cross-modal data registration, attention mechanism, visual recognition and understanding, and embodied control. The model comprises 2 significant parts of self-driving interactive cognition: perceptual intelligence and behavioral intelligence. In particular, behavioral intelligence is to learn an excellent driving experience via the driving brain of self-driving vehicles, which reflects the analysis of humanoid driving and makes correct decision control over the vehicle, which not only is a good analysis of interactive cognition but also can effectively improve the comfort, satisfaction, safety, intelligence, and efficiency in vehicles.

#### Analysis matrix of self-driving interactive cognition

**Fig. 9.**
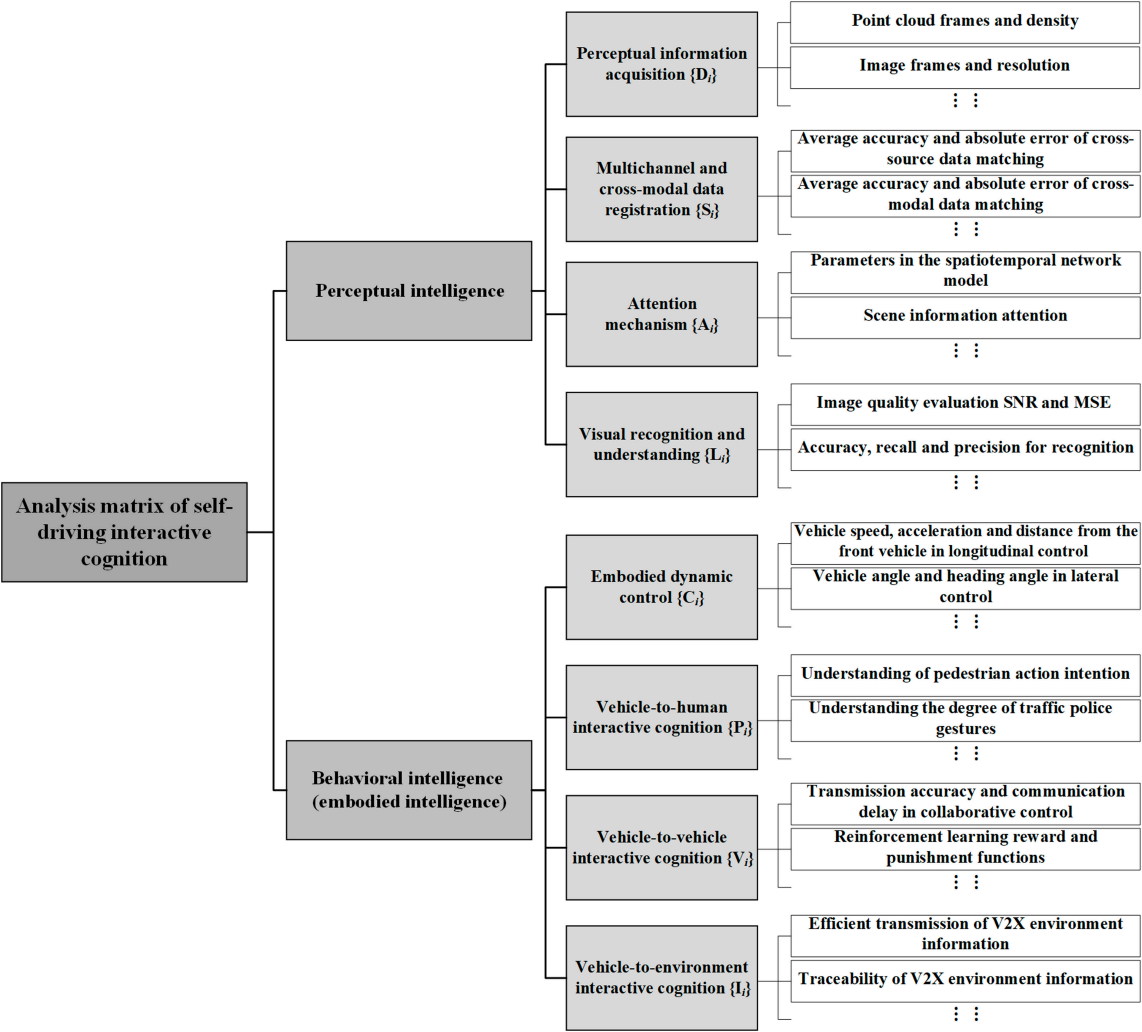
Analysis matrix of self-driving interactive cognition. SNR, signal-to-noise ratio; MSE, mean-square error; V2X, Vehicle to Everything.

**Fig. 10.**
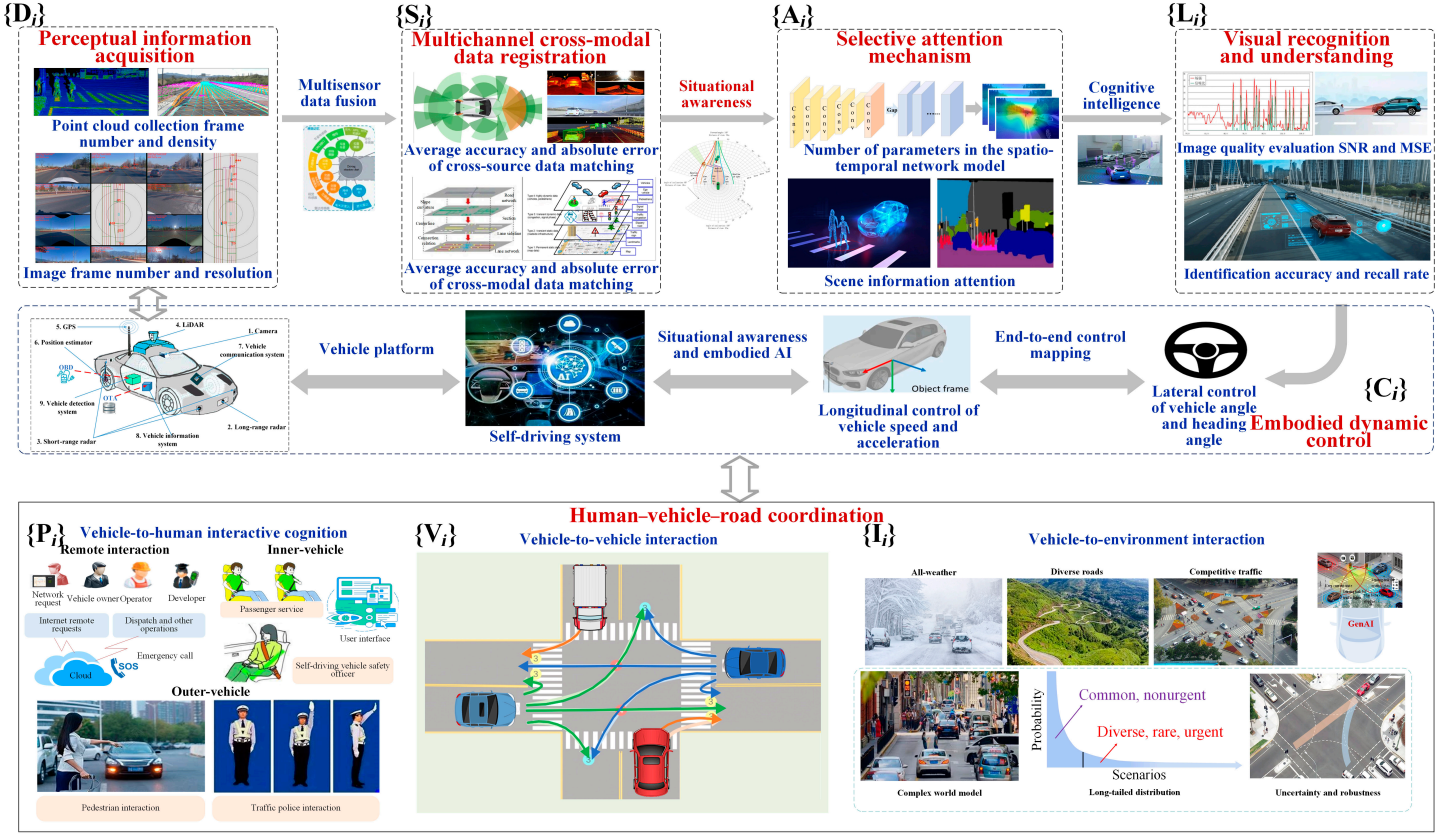
Multidimensional analysis model of self-driving interactive cognition. GPS, Global Positioning System; AI, artificial intelligence; GenAI, generative AI; OBD, onboard diagnostics; OTA, over-the-air.

Considering vehicle’s embodied intelligence as an essential basis, we first establish an analysis matrix of interactive cognition to achieve humanoid driving analysis. From the perspective of perceptual intelligence, the following dimensions are included: the analysis of self-driving vehicle sensors such as cameras, radar, and navigation to obtain perceptual data $\left\{D_{i}\right\}$; the analysis of multichannel and cross-modal data registration $\left\{S_{i}\right\}$; the analysis of the attention mechanism for perceived information $\left\{A_{i}\right\}$; and the analysis of visual recognition and understanding $\left\{L_{i}\right\}$. Behavioral intelligence incorporates the following dimensions: the analysis of steering, braking, and acceleration of their own vehicle with embodied control $\left\{C_{i}\right\}$; the analysis of body language interaction between vehicles and humans $\left\{P_{i}\right\}$; the analysis of vehicle language interactive cognition $\left\{V_{i}\right\}$; and the analysis of synergistic interactive cognition between vehicles and environments $\left\{I_{i}\right\}$, thus forming the analysis matrix of self-driving interactive cognition, as shown in Fig. 9. According to the analysis matrix of self-driving interactive cognition, a multidimensional analysis model of self-driving interactive cognition is further constructed, as shown in Fig. 10.

#### The multidimensional analysis model of interactive cognition based on nonlinear CRITIC–TOPSIS

The TOPSIS method, also known as the superior and inferior solution distance method, was proposed by Yoon and Hwang [32] to evaluate and rank the proximity of a limited number of evaluation objects to the ideal target. This method fully uses the original data, and the results accurately reflect the differences between the objects in the analysis model. In addition, data calculation is convenient and feasible because the data distribution and sample content are not strictly limited. However, the conventional TOPSIS method defaults to the same weight for each indicator. The importance of each index is inconsistent in the actual situation, making the existing TOPSIS method unsuitable for evaluating multiple index objects with different weights. The TOPSIS method based on entropy rights can calculate the weight of each index according to the dispersion degree of the data in each index and complete the task of comprehensive evaluation of multiple indexes.

In the analysis model of self-driving interactive cognition, however, there is a correlation between the indexes. For example, the accuracy of visual recognition and understanding $\left\{L_{i}\right\}$ is affected by the quality of perceptual information acquisition $\left\{D_{i}\right\}$. Therefore, the TOPSIS method based on entropy rights has limitations in evaluating the analysis model of self-driving interactive cognition due to its incompetence in calculating the correlation between the indexes. The CRITIC method was proposed by Diakoulaki et al. [33] as an objective weighting method based on data volatility. This method, combined with the correlation coefficient and information weight, reflects the index information quantity based on the contrast intensity of the evaluation index and the conflict between the indexes. More information will be reflected if the contrast intensity and conflicts are more significant, and the weight of the corresponding index will be greater. As a result, it can fully reflect the correlation between the indexes in multi-index evaluation tasks. Nevertheless, the traditional CRITIC method [33] based on the Pearson correlation coefficient is limited to measuring the linear correlation between the indexes. The evaluation of the analysis model of self-driving interactive cognition is a complex nonlinear process, and the calculation and statistical methods of indicators are different. Therefore, it is difficult for the existing CRITIC method to accurately evaluate the analysis of self-driving interactive cognition. In this paper, we innovatively propose the nonlinear-CRITIC–TOPSIS method based on Spearman’s rank correlation coefficient, considering both indicator correlation and nonlinear features.

In the process of self-driving interactive cognition, the interactive cognition of self-driving based on the Wiener model forms situation map clusters, including the perceptual graph clusters of $\left\{D_{i}\right\}$, $\left\{P_{i}\right\}$, $\left\{S_{i}\right\}$, $\left\{A_{i}\right\}$, and $\left\{L_{i}\right\}$ as well as the behavioral graph clusters of $\left\{C_{i}\right\}$, $\left\{P_{i}\right\}$, $\left\{V_{i}\right\}$, and $\left\{I_{i}\right\}$. The analysis model of self-driving interactive cognition forms the octuple according to situation graph clusters. We based the multidimensional analysis model of self-driving interactive cognition on the nonlinear-CRITIC–TOPSIS method as follows:

1. Determine the index set of self-driving interactive cognition analysis.

$$U=D_{i},S_{i},L_{i},A_{i},C_{i},P_{i},V_{i},I_{i}$$ (16)

where U denotes the set of interactive cognition analysis indicators for self-driving vehicles.

2. Determine the set of objects assessed for self-driving interactive cognition analysis.

$$V=D_{\mathrm{ij}},S_{\mathrm{ij}},L_{\mathrm{ij}},A_{\mathrm{ij}},C_{\mathrm{ij}},P_{\mathrm{ij}},V_{\mathrm{ij}},I_{\mathrm{ij}}$$ (17)

where V denotes the evaluation object of the octuplet set of the self-driving interactive cognition analyses.

3. Determine the total assessment scores of self-driving interactive cognition.
  (1) Build the raw data matrix. The raw data matrix R is formed according to the *m* evaluated objects and *n* indicators.

R=R1R2…Rn=rijm×n=r11⋯r1j⋯r1n⋮⋮⋮⋮⋮ri1⋮rij⋮rin⋮⋮⋮⋮⋮rm1⋯rmj⋯rmn (18)

where column *j* in R represents the raw data of the *j*th index of the *j*th evaluated object.

(2) Standardize the initial data matrix $R={\left(r_{\mathrm{ij}}\right)}_{m\times n}$.

R′=rij′m×n=rij−minrijmaxrij−minrijpositiveindicatormaxrij−rijmaxrij−minrijnegativeindicator=r11′⋯r1j′⋯r1n′⋮⋮⋮⋮⋮ri1′⋮rij′⋮rin′⋮⋮⋮⋮⋮rm1′⋯rmj′⋯rmn′ (19)

(3) Compute the volatility $S_{j}$ of the data within each index.

$$S_{j}=\sqrt{\frac{\sum_{i=1}^{m}{\left[r_{\mathrm{ij}}^{\prime }-\text{mean}\left(r_{j}^{\prime }\right)\right]}^{2}}{n-1}}$$ (20)

where $\text{mean}\left(r_{j}^{\prime }\right)$ represents the mean value of the data of each indicator (column) after standardization and $S_{j}$ is the standard deviation.

(4) Calculate the conflict between the indicators.
1) Calculate Spearman’s correlation coefficient $\rho_{\mathrm{tj}}$ between indicators.

$$\rho_{\mathrm{tj}}=\frac{\sum_{i=1}^{m}\left[r_{\mathrm{it}}^{\prime \prime }-\text{mean}\left(r_{t}^{\prime \prime }\right)\right]\cdot \left[r_{\mathrm{ij}}^{\prime }-\text{mean}\left(r_{j}^{\prime }\right)\right]}{\sqrt{\sum_{i=1}^{m}{\left[r_{\mathrm{it}}^{\prime \prime }-\text{mean}\left(r^{\prime \prime }\right)\right]}^{2}\cdot \sum_{i=1}^{m}{\left[r_{\mathrm{ij}}^{\prime \prime }-\text{mean}\left(r^{\prime \prime }\right)\right]}^{2}}}$$ (21)

where $r_{\mathrm{ij}}^{\prime \prime }$ is the rank of the data corresponding to the *j*th index of the *i*th evaluated object in the standardized matrix and $\text{mean}\left(r_{t}^{\prime \prime }\right)$ and $\text{mean}\left(r_{j}^{\prime \prime }\right)$ represent the mean rank of the data in the standardized row *t* and *j*, respectively.

2) Calculate the conflict between the indicators $B_{j}$.

$$B_{j}=\sum_{t=1}^{n}\left(1-\rho_{\mathrm{tj}}\right)$$ (22)

(5) Calculate the amount of information for each indicator $\;D_{j}$.

$$D_{j}=S_{j}\cdot B_{j}$$ (23)

(6) The weight of the index is calculated according to the information quantity of each index $w_{j}$.

$$w_{j}=\frac{D_{j}}{\sum_{j=1}^{n}D_{j}}$$ (24)

(7) The weighted standardized matrix $Z_{\mathrm{ij}}$ is obtained from the standardization matrix $R^{\prime }$ and each index weight $w_{j}$.

$$Z_{\mathrm{ij}}=w_{j}R^{\prime }$$ (25)

(8) The composite score $C_{i}$ of each evaluated object was calculated based on the gap between each index and the positive and negative ideal solution.

$$\begin{array}{c} \begin{array}{c} \begin{array}{c} C_{i}=\frac{\sqrt{\sum_{j=1}^{n}{\left(\min\left\{z_{1j},\;z_{2j},\;\ldots ,\;z_{\mathrm{mj}}\right\}-z_{\mathrm{ij}}\right)}^{2}}}{\sqrt{\sum_{j=1}^{n}{\left(\max\left\{z_{1j},\;z_{2j},\;\ldots ,\;z_{\mathrm{mj}}\right\}-z_{\mathrm{ij}}\right)}^{2}}+\sqrt{\sum_{j=1}^{n}{\left(\min\left\{z_{1j},\;z_{2j},\;\ldots ,\;z_{\mathrm{mj}}\right\}-z_{\mathrm{ij}}\right)}^{2}}} \end{array} \end{array} \end{array}$$ (26)

where the value range of $C_{i}\in \left[0,\;1\right]$, $\max\left(z_{1j},\;z_{2j},\;\cdots z_{\mathrm{mj}}\right)$ is the positive ideal of each index, and $\min\left(z_{1j},\;z_{2j},\;\cdots z_{\mathrm{mj}}\right)$ is the negative ideal solution of each index. As a result, the calculated combined score results can further analyze the capability of self-driving interactive cognition.

## Data Availability

This study utilizes both public and self-constructed datasets. The public datasets introduced in the “Public datasets” section are freely available at their official repositories. The self-constructed dataset described in the “Self-collected multiview pedestrian action and traffic police gesture dataset” section can be accessed via the following link: http://www.mananlab.tech/datasets.
